# Clinical Outcome and Predictors of Customized Polyetheretherketone Post‐and‐Cores for Residual Root and Crown: A Retrospective Study

**DOI:** 10.1002/cre2.70066

**Published:** 2025-02-18

**Authors:** Xin Wang, Chen Liu, Yuchen Liu, Dan Ma, Ruifang Ren, Mingxing Zhang, Jiawen Guo, Yimin Zhao, Dongmei Li, Shizhu Bai

**Affiliations:** ^1^ State Key Laboratory of Oral & Maxillofacial Reconstruction and Regeneration, National Clinical Research Center for Oral Diseases, Shaanxi Key Laboratory of Stomatology, Digital Dentistry Center, School of Stomatology The Fourth Military Medical University Xi'an Shaanxi China; ^2^ Hospital of Stomatology, Guangdong Provincial Key Laboratory of Stomatology, Guanghua School of Stomatology Sun Yat‐sen University Guangzhou China

**Keywords:** customized post‐and‐cores, polyetheretherketone, retrospective study, treatment outcome

## Abstract

**Objectives:**

This retrospective, non‐interventional study aimed to evaluate the longevity and factors influencing the success of custom‐fabricated polyetheretherketone (PEEK) post‐and‐cores in endodontically treated teeth (ETT).

**Material and Methods:**

During the observation period (2019–2022), 63 patients received 100 customized PEEK post‐and‐cores. Clinical outcomes were analyzed based on the following parameters: age, gender, tooth type, tooth position, proximal contacts, remaining cavity walls, antagonist contacts, and final restoration. Kaplan–Meier analysis was used for the survival probability. Log‐rank tests were used to identify univariate associations between failure rates and other potential factors. Cox regression was used to assess the risk of failure and determine possible risks between the identified factors at a cut‐off point *p*‐value (0.25 in univariate analysis).

**Results:**

The median follow‐up duration was 20.62 months, with a maximum of 40.57 months. 93 restorations were judged as successful and 99 teeth survived. The main failure type was decementation of the restoration (*n* = 4). The annual failure rate was 3.4%. Gender, remaining tooth structure, and final restoration were found to be correlated with success rates in the preliminary univariate analysis (Log‐rank tests) to determine the association between failure rates and potential factors. Multifactorial survival analysis (Cox regression) showed that teeth with coronal walls had a significantly lower failure risk than deprived teeth, even with the ferrule effect. (HR = 0.04; 95% CI for HR = 0.01–0.29; *p* = 0.002).

**Conclusions:**

PEEK post‐and‐cores adapt well to clinical restorative needs and offer favorable short‐term clinical outcomes. The remaining cavity wall was a significant success rate predictor.

## 1 Introduction

When the remaining available tooth structure lacks the height to form an adequate full crown retention pattern or provide adequate resistance, post‐and‐cores restoration becomes a commonly recommended approach to ensure retention and support for the final full crown restoration (Qing et al. 2007). The cervical region of natural teeth is recognized as a stress concentration area, and any defect in this region can substantially diminish the tooth's fracture resistance (Scholz et al. 2022). However, post‐and‐cores serve to mitigate the stress concentration by redistributing it to other regions of the tooth, consequently reinforcing the fracture resistance of the abutment tooth (Skupien et al. 2015; Jurema et al. 2022). Notably, the material type and design of the post affect the fracture resistance of the endodontically treated teeth as well as the retention of the post, which in turn relates to the long‐term restorative outcome (Coelho et al. 2009).

The mechanical properties of the ideal material of post‐and‐cores should be similar to those of dentin (Fernandes, Shetty, and Coutinho 2003), which in turn promotes the uniform distribution of occlusal forces to the tooth tissue and restoration, thereby diminishing the risk of irreparable root fracture (Yu et al. 2022). However, conventional post materials such as metals, ceramics, and fibers typically possess a higher modulus of elasticity than dentin. This discrepancy can potentially elevate the likelihood of stress concentration in the tooth tissue and increase the risk of root fractures (Yu et al. 2022).

Prefabricated posts offer notable advantages in terms of their mechanical properties, biocompatibility, and esthetics. These serve to protect the patient's roots. Since the 1990s, prefabricated posts have gained widespread application in clinical practice. When coupled with appropriately matched canal preparation instruments, they yield a more satisfactory cementation effect. Nevertheless, a notable drawback is their limited adaptability to the root canal (Sb et al. 2018), leading to reduced long‐term efficacy, particularly in cases involving oval and flared root canals (Awad and Marghalani 2007). Additionally, the use of prefabricated posts increases the number of additional cementation interfaces, which may increase the restoration failure rate (Vano et al. 2006).

The challenges posed by the high modulus of elasticity associated with traditional materials and the limitations of prefabricated posts have been effectively addressed through precision milling of customized polyetheretherketone (PEEK) post‐and‐cores using computer‐aided design and computer‐aided manufacturing (CAD‐CAM) technology. As a high‐performance thermoplastic polymer, PEEK has been introduced into the field of dentistry in recent years because of its exceptional biocompatibility, mechanical strength, vibration‐damping properties, radiation resistance, chemical stability, and heat resistance (Kurtz and Devine 2007; Zol et al. 2023; Liu et al. 2022). Compared with conventional post materials, the elastic moduli of PEEK and its variants are considerably similar to those of dentin, resulting in a more favorable stress distribution within the tooth structure, particularly benefiting weakened roots (Gontijo et al. 2022). The restoration of PEEK post‐and‐cores exhibits a more repairable postfracture when subjected to excessive occlusal loads (Carvalho et al. 2018). PEEK can be manufactured using various techniques, including casting under heat and pressure, milling, and 3D printing (Zoidis and Papathanasiou 2016). The milled customized post‐and‐cores provide passivity of fit and are well adapted to noncircular root canals, consequently increasing the adhesive strength and fracture resistance of the tooth tissue (Güven et al. 2020; Costa et al. 2022). Moreover, it is preferred for teeth that require alteration of the tooth's long axis by changing the angle of the core (Martino et al. 2020).

Numerous finite‐element experiments and in vitro studies have explored the properties of PEEK post‐and‐core in dentistry. However, a notable gap exists in clinical studies assessing the tangible therapeutic effectiveness of PEEK post‐and‐core in dental practice. Considering the absence of substantial clinical evidence guiding the selection of optimal materials for post‐and‐core restorations, the purpose of this retrospective clinical study was to evaluate the clinical outcomes of PEEK post‐and‐cores in cases of residual roots and crowns. In addition, this research aimed to investigate the significance of potential factors that could influence the survival rates of these restorations.

## 2 Materials and Methods

### 2.1 Study Design

Patients who underwent PEEK post‐and‐core treatment after endodontic therapy at the Third Affiliated Hospital of the Fourth Military Medical University between December 2019 and December 2022 were examined for pertinent data. The basic data of patients who met the inclusion criteria were collected. Clinical examinations and radiographic analyses were performed to determine the success rate and functional state of the PEEK posts.

In accordance with the 2008 Declaration of Helsinki (updated in 2013), all participants signed an informed consent form and underwent follow‐up and radiographic examinations.

This study was approved by the Ethics Committee of the Third Affiliated Hospital of the Fourth Military Medical University (Approval Number: IRB‐REV‐2022188). It has been registered in the Chinese Clinical Trial Registry (https://www.chictr.org.cn/) and has been approved (Approval Number: ChiCTR2200066855).

### 2.2 Patient Selection

#### 2.2.1 Inclusion Criteria

Eligible participants for this study encompassed both males and females meeting the following criteria:

- Age of 18 years or older with generally normal occlusal relationships.
- At least one tooth underwent endodontic treatment and required post‐and‐core restoration between December 2019 and December 2022, followed by treatment with a PEEK post‐and‐core.
- Proper oral hygiene was maintained, with a Full Mouth Plaque Score (FMPS) of less than 15%.
- Be in general good health without severe smoking, alcohol consumption, or any other adverse habits.

#### 2.2.2 Exclusion Criteria

Patients meeting any of the following criteria, as reported in their medical records, were excluded from the study:

- Untreated periodontal disease or a predisposition to dental caries.
- Presence of adverse oral habits such as bruxism or teeth clenching.
- History of a suspected or diagnosed cracked tooth.
- Inability or unwillingness to provide informed consent.

### 2.3 Clinical Procedure

**Figure 1:**
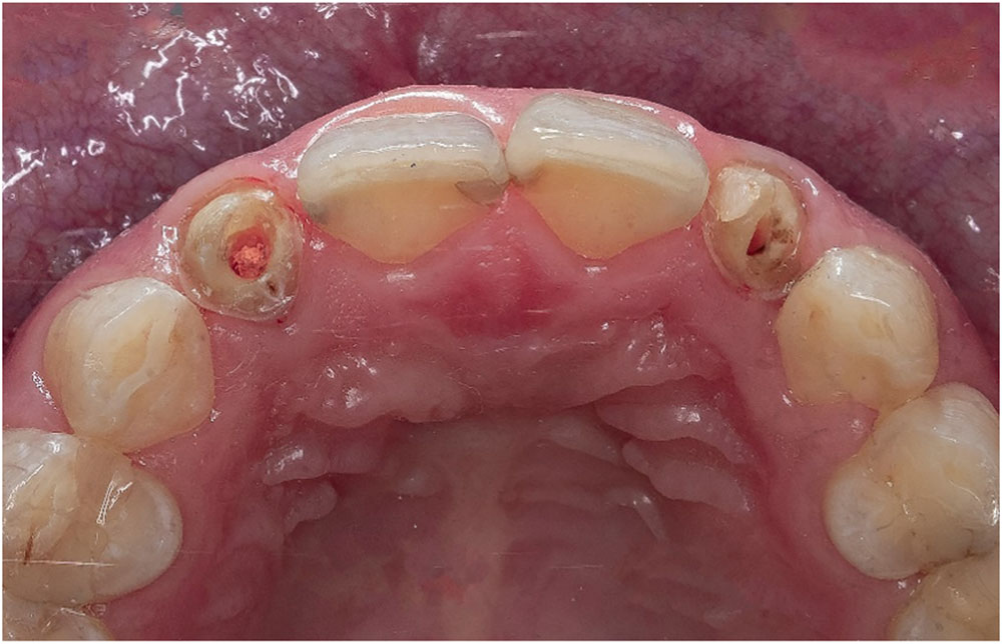
Intraoral morphology preparation of posterior teeth.

All patients underwent endodontic treatment, and prosthodontists with 20 years of clinical experience determined whether the restoration of post‐and‐core was necessary for all patients. The post space was prepared using post drills (Cytec; Hahnenkratt, Germany), gradually enlarging the post space to an appropriate size based on the root canal diameter while removing any remaining root canal filling material from the canal walls with an ultrasonic tip (Instrument Nr.; Dentsply Sirona). A minimum of 4.0 mm of root filling material was left at the root apex. The post‐space diameter was approximately one‐third of the root diameter after root canal preparation, and the post‐space morphology matched the cross‐sectional morphology of the roots. The abutment tooth was prepared with the shoulder to fit the prosthetic restoration, followed by the refinement of the cervical shoulder and polishing (Figure 1).

**Figure 2:**
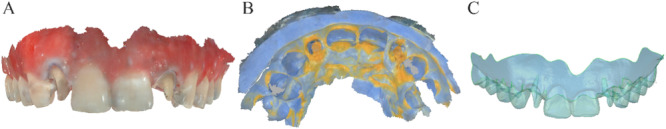
Digital fusion model, (A) Digital tooth arch model, (B) Digital impression model, (C) Fusion of tooth arch from model A and post space from model B into a complete digital model.

The dental arch was thoroughly dried, and an intraoral scanner (Aoralscan 3; Shining 3D, Hangzhou, China) was used to scan the abutment tooth opposing the dental arch and occlusal relationship. A two‐step impression was taken of the tooth being treated and the two adjacent tooth positions with scannable polyvinylsiloxane (PVS) silicone (Honigum Pro Light and Heavy Scan; DMG Dental GmbH, Hamburg, Germany). The redundant silicon was trimmed to expose the scanning field. The impression was then digitalized with the intraoral scanner to obtain a three‐dimensional digital model of the morphology of the post‐space. Reverse engineering software (Geomagic Wrap 2021; 3D Systems, North Carolina, USA) was used to integrate the post‐space partial digital model with a digital dentition model that contained occlusal information (Figure 2). The digitalized data were imported into dental design software (Dental System 19; 3Shape A/S, Copenhagen, Denmark) for the post‐and‐core crown design. First, the margin line of the post and crown was delineated, followed by the design of the post's insertion direction, refinement of the core's morphology, and, ultimately, the design of the prosthetic restoration surrounding the post‐and‐core. Subsequently, the digital post‐and‐core data designed were exported to the 5‐axis milling machine (Ceramill Matik; Amann Girrbach, Koblach, Austria) for the fabrication of the PEEK (BioPAEK, Sino‐Dentex Co. Ltd. Changchun, China) post‐and‐core.

**Figure 3:**
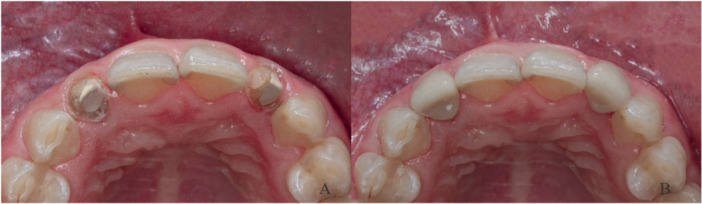
(A) Trial placement of post‐and‐cores, (B) Intraoral morphology after cementation of restoration.

Before bonding, rubber dam isolation and a saliva ejector were used. A trial fitting of the crown was conducted both with and without the post. The crown fits smoothly in both scenarios, indicating that the post was properly seated. Subsequently, a meticulous cleaning of the post space was conducted using a 2.5% sodium hypochlorite solution, followed by thorough rinsing and drying using air and paper points. Both the PEEK post and full crown were cleaned with 75% ethanol and air‐dried. Subsequently, the surfaces were prepared for cementation by applying an adhesive (Visio.link; Bredent, Senden, Germany) and curing for 90 s. The post space was etched with 35% phosphoric acid for 15 s, followed by rinsing and air drying. Cementation was performed according to the manufacturer's instructions. A universal adhesive (Single Bond Universal Adhesive, 3 M ESPE, St. Paul, Minnesota) was used in combination with resin adhesive (RelyX Ultimate Adhesive Resin Cement, 3 M ESPE, St. Paul, Minnesota), ensuring a simultaneous cementation of the post and crown. A final curing step was performed for 40 s (Figure 3). Radiographs were captured after cementation to confirm the success of the procedure. Upon completion of the restoration, the patient received detailed oral hygiene instructions.

### 2.4 Data Collection

In March–April 2023, the baseline data of the patients were examined and recorded by prosthodontists with over 5 years of experience. This data included the patient's age (< 50/ ≥ 50), gender (female/male), observation period (date of cementation/date of the final observation or failure), position of tooth (maxilla/mandible, anterior/posterior teeth), the number of remaining tooth structure walls (none/≥ 1), proximal contacts (0,1/2), antagonist contacts (periodontal/non‐periodontal supported), and final restoration (single crown/fixed bridge/combined fixed‐removable).

Prosthodontists with at least 10 years of clinical experience examined the crowns without knowing the patient's circumstances. A dental probe and mirror were used to examine the secondary caries, marginal gaps, mobility, and pain around the restorations, with subsequent evaluation of success or failure.

Periapical radiographs were obtained using a dental imaging processing system (Denoptix QST Digital Imaging System, Dentsply). These radiographs were then submitted to an oral radiologist for the assessment of periapical lesions, periodontal membrane thickness, fit between the post and tooth structure, post fractures, root fractures, and secondary caries.

The collection of the abovementioned information and examination of the restorations were conducted independently by two dentists. In cases of disagreement, a third assessment was conducted by both dentists to reach a consensus.

### 2.5 Evaluation Criteria

The criteria for failure included secondary caries, root fracture (vertical or horizontal), loss of post‐retention, loss of crown retention, post‐and‐core fracture, and endodontic problems. Among these, the ultimate outcome of tooth extraction was considered absolute failure, whereas failure meeting the indications for restoration was categorized as relative failure. Success was defined as the absence of absolute and relative failure, whereas survival was defined as the absence of absolute failure.

### 2.6 Statistical Analysis

Descriptive analysis was conducted by calculating the frequency and proportion of categorical variables and evaluating the success and survival rates of PEEK posts. The time to restoration failure, measured in months from the completion of the post‐and‐core to the date of failure, was assessed. The annual failure rates (AFR) of the restorations were computed using the formula (1 − *y*)^*z*^ = (1 − x), where *y* represents the mean AFR and *x* represents the total failure rate at *z* years, taking into account the survival data for all restorations (Sarkis‐Onofre et al. 2020). Kaplan–Meier analysis was employed to illustrate the cumulative success rate of PEEK post‐and‐core in the overall sample. Log‐rank tests were used to compute the failure rates across different baseline characteristics, initially identifying univariate associations between failure rates and other potential factors. The Cox proportional hazards model was used in multivariate analysis to determine possible risks between identified factors at a cutoff point *p*‐value (0.25 in univariate analysis). Hazard ratios (HR) and two‐sided 95% confidence intervals (CI) were derived. All data were processed using SPSS 25 (IBM Corp., Armonk, NY, USA) with a two‐tailed α level of 0.05. Statistical significance was set at *p* < 0.05.

## 3 Results

Patients meeting the inclusion and exclusion criteria amounted to 63 individuals, representing a total of 100 post‐and‐core restorations. Among them, 29 patients (46.0%) were male and 34 were female (54.0%). The average age was 54.12 ± 18.09 years, with a median follow‐up duration of 20.62 months (IQR 13.55–25.69).

Failure rate of post‐and‐core restorations: Among the 100 post‐and‐core restorations, seven failed, resulting in a total failure rate of 7% and an annual failure rate (AFR) of 3.4%. Among these, one absolute failure occurred, accounting for a failure rate of 1% and an AFR of 0.3%. Six relative failures occurred, with a failure rate of 6% and an AFR of 2.0%. The primary reasons for restoration failure were post‐and‐core decementation (*n* = 4), periapical periodontitis (*n* = 2), and tooth fracture (*n* = 1). A root fracture (*n* = 1) occurred in the maxillary central incisor, and the tooth did not meet the criteria for restoration, leading to extraction. Basic information on failed post‐core restorations is presented in Table 1.

**Table 1:** Characteristics of each failure observed in the study.

Position	Time of failure (m)	Age	Gender	Proximal contacts	Remaining cavity walls	Final restoration	Failure reason
23	13.67	≥ 50	Female	2	0	Single crown	Crown and post‐debonding
12	18.40	< 50	Male	2	4	Fixed bridge	Endodontic failure
34	19.30	≥ 50	Female	2	0	Fixed bridge	Crown and post‐debonding
21	21.57	< 50	Female	2	0	Single crown	Root fracture
23	27.93	≥ 50	Female	2	2	Single crown	Crown and post‐debonding
15	25.20	< 50	Female	2	0	Combined fixed‐removable	Endodontic failure
11	22.23	≥ 50	Male	2	0	Single crown	Crown and post‐debonding

**Figure 4:**
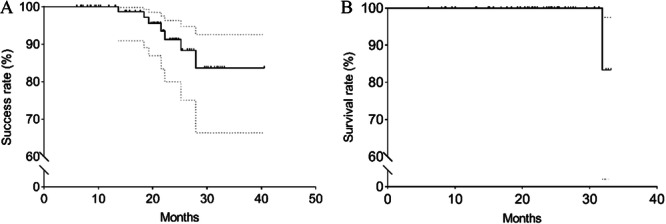
Kaplan–Meier estimates considering the success (A) and survival rates (B).

Kaplan–Meier survival graphs and log‐rank test: the success and survival rates of PEEK post‐and‐core restorations were assessed using Kaplan–Meier analysis, as illustrated in Figure 4. Of the 93 restorations, they were considered successful (cumulative success rate, 83.70%), and 99 restorations were deemed to have survived (cumulative survival rate, 87.50%). Preliminary associations between failure rates and various baseline characteristics were determined using the log‐rank test, with specific data provided in Table 2. Baseline characteristics with a *p*‐value less than 0.25 included gender, final restoration, and remaining tooth structure.

**Table 2:** Frequency, number of failures, and mean success time included in the study, as well as log‐rank test of time until failure by category of each baseline characteristic.

Category	Frequency [*n* (%)]	Failures [*n* (%)]	Mean success time (months)	HR	95%CI	*p* value
**Age**						
< 50	42 (42%)	3 (7%)	21.20	1.0	Reference	
≥ 50	58 (58%)	4 (7%)	19.64	1.4	0.3–6	0.669
**Gender**						
Female	47 (47%)	5 (11%)	19.47	1.0	Reference	
Male	53 (53%)	2 (4%)	21.02	0.3	0.1–1.3	0.120
**Tooth type**						
Incisors/canines	60 (60%)	5 (8%)	20.86	1.0	Reference	
Premolars/molars	40 (40%)	2 (5%)	19.45	0.7	0.2–3.3	0.672
**Position of tooth**						
Maxillary	69 (69%)	6 (9%)	20.06	1.0	Reference	
Mandible	31 (31%)	1 (3%)	20.81	0.4	0.1–2.1	0.390
**Proximal contacts**						
0,1	18 (18%)	0 (0%)	18.53	1.0	Reference	
2	82 (82%)	7 (9%)	20.68		−1.0 to −1.0	0.285
**Remaining cavity walls**						
None	19 (19%)	5 (26%)	20.79	1.0	Reference	
≥ 1	81 (81%)	2 (2%)	20.18	0.1	0–0.6	0.001
**Antagonist contacts**						
Periodontal supported	88 (88%)	7 (8%)	20.53	1.0	Reference	
Non‐periodontal supported	12 (12%)	0 (0%)	18.56	0	−1.0 to −1.0	0.444
**Final restoration**						0.306
Single crown	34 (34%)	4 (12%)	18.61	1.0	Reference	
Fixed bridge	41 (41%)	2 (8%)	22.26	0.3	0.1–1.6	0.152
Combined fixed‐removable	25 (25%)	1 (4%)	19.36	0.4	0–3.2	0.365

Abbreviations: CI, confidence interval; HR, Hazard‐Ratios.

Cox regression analysis: In the Cox proportional hazards model (Table 3), a significant correlation was observed between the number of remaining tooth structure walls and the failure rate among the investigated factors (HR = 0.04; 95% CI for HR = 0.01–0.29; *p* = 0.002).

**Table 3:** Multivariate Cox proportional hazards regression analysis of time until failure as a function of baseline characteristics.

Category	HR	95% CI	*p* value
**Gender**			
Female	1.00	Reference	
Male	0.29	0.05–1.64	0.162
**Remaining cavity walls**			
None	1.00	Reference	
≥ 1	0.04	0.01–0.29	0.002
**Final restoration**			0.074
Single crown	1.00	Reference	
Fixed bridge	0.13	0.02–0.85	0.033
Combined fixed‐removable	0.15	0.01–1.63	0.119

Abbreviations: CI, confidence interval; HZ, hazard ratios.

## 4 Discussion

To our knowledge, this is the first retrospective clinical trial to assess the success rate of PEEK post‐and‐core restorations. The data were retrospectively acquired using a standardized protocol. Since 2019, all baseline patient information and digital impressions before treatment have been stored in the Digital Center database.

In recent years, most scholars have primarily focused their research on PEEK, a novel restorative material, in terms of in vitro experiments, with limited case reports and clinical studies available. Currently, few case reports have described the clinical application of PEEK post‐and‐core restorations (Zoidis 2021; Galgali et al. 2021; Kasem, Shams, and Tribst 2022). Therefore, the results of this clinical study provide credible references for future clinical applications of PEEK post‐and‐core restorations.

Because of variations in the design and procedures of different clinical trials, comparability with other studies should be considered problematic. Therefore, the results are compared with those of other studies for reference. To date, no comparable data for PEEK post‐and‐cores in endodontically treated teeth are available. In a prospective study over 2 years (24–27 months), the failure rate of glass fiber posts was 12% (Naumann, Blankenstein, and Dietrich 2005). The overall failure rate after 3 years of using carbon fiber posts reached 13% (Mannocci et al. 2002). For cast metal posts repairing different tooth positions after 1 year, the overall failure rate ranged from 5% to 9% (Bergoli et al. 2023). In this study, the overall failure rate of PEEK was 7%, which was slightly lower than that reported in other studies.

In this study, only one absolute failure occurred, resulting in a failure rate of 1%, which was lower than the success rates reported in previous studies that used other materials for post‐and‐core restorations. Sarkis‐Onofre et al. compared the success rates of cast metal posts and glass fiber posts in cases where none of the treated teeth had a remaining coronal tooth structure; after 5 years, 10 out of 183 restorations experienced root fractures (Sarkis‐Onofre et al. 2020). Bergoli et al. used glass fiber posts for restorations, with an average follow‐up time of 63 months, and observed 9 root fractures out of 142 posts (Bergoli et al. 2023). Rafael et al. used glass fiber and metal posts for restorations, with follow‐up time of 3 years, and observed 2 root fractures out of 72 posts (Sarkis‐Onofre et al. 2014). Stress concentration and stress fatigue are among the primary reasons for tooth structure fractures. PEEK posts have a similar elastic modulus to tooth structure, and when bearing occlusal forces, they do not create stress concentrations on the remaining tooth structure surface. Stress is evenly distributed on both the post‐and‐core and tooth structure surfaces, reducing the load borne by the remaining tooth structure and thus decreasing the risk of root fractures (Gontijo et al. 2022; Carvalho et al. 2018). Combined with the results of the clinical experiments, PEEK post‐and‐cores were found to perform better than in other studies in maintaining the residual tooth structure during the brief follow‐up period. In a preliminary univariate analysis to determine the association between failure rates and potential factors, gender, amount of remaining tooth structure, and final restoration were found to correlate with success rates. The Cox model revealed a significant correlation between the amount of remaining tooth structure and survival rate. The interaction between factors such as the preparation of the canal space and the quantity of coronal and root dentin remaining after root canal treatment has been recognized as critical for the survival of pulpless teeth (Faria et al. 2011). Restorations without a dentin ferrule may be more prone to failure compared to those with one or more dentin ferrules (Cagidiaco et al. 2008). Therefore, the importance of maintaining a 1.5–2 mm minimum dentin ferrule for mechanical properties has been emphasized, especially for enhancing the longevity of endodontically treated teeth restored with posts and crowns (Nr and Pr 2002; Soares et al. 2012). In this study, among the 19 restorations without errors, five failed, resulting in a failure rate of 26%. In a 3‐year clinical study, out of 60 fiber restorations without a dentin ferrule, 29 failures occurred, leading to a failure rate of 48.3% (Cagidiaco et al. 2008). For the restoration of teeth without ferrule, this study suggests that personalized PEEK posts with a lower elastic modulus may be a better choice for restoration in the short‐term period.

Compared with fixed bridges, post‐and‐cores with single crowns had a 7.7 times higher risk of failure, showing a significant difference. This may be related to the inability of single crowns to effectively distribute occlusal forces. Single‐crown and combined fixed‐removable restorations did not differ significantly in terms of failure risk. In a 16‐year follow‐up study, Vogler et al. suggested that post‐and‐cores under combined fixed‐removable restorations had a 2.5 times higher risk of failure than single crowns (Vogler, Lehmann, Rehmann, et al. 2022). The authors attributed this to the lower success rate of post‐and‐cores abutments for removable partial dentures, which could be related to the occlusal balance between the abutments and the remaining ridge.

Decementation remained a primary factor in post‐and‐core failures in this study, with a detachment rate of 4%, accounting for 57.14% of all failures. However, PEEK presents a challenge in terms of adhesion owing to its low surface energy and chemical inertness (Zol et al. 2023). In this study, before cementation, the PEEK post‐and‐cores underwent sulfuric acid etching, sandblasting, and Visio.link adhesive pretreatment to achieve a higher adhesive strength, as reported in previous studies (Schmidlin et al. 2010). Nonetheless, several factors influence the cementing effect. In this study, decementation post‐and‐cores were reprepared and cemented, and no further decementation was observed within the observation period. Vogler, Lehmann, Schlenz, et al. (2022) conducted a survival analysis of recementation post and crown restorations, and concluded that initial decementation might not affect the retention of the post‐and‐core. Only in cases involving fixed combined removable prosthetic restorations, it may be necessary to reduce the occlusal force applied to the post‐and‐cores to improve the cementing efficiency. The aforementioned discussions collectively suggest that the preservation of the remaining tooth structure can be realized, provided there are no occurrences of absolute failure. Therefore, PEEK post‐and‐cores have the potential to meet clinical restorative needs and exhibit promising prospects for applications.

PEEK has a relatively short history in restorative dentistry and requires clinical observation to evaluate short‐term clinical outcomes. However, extended follow‐up observations are imperative to comprehensively assess the long‐term effectiveness. The research began with the first PEEK post‐and‐core restoration in December 2019 during the COVID‐19 pandemic, which led to difficulties for patients seeking treatment, resulting in a limited number of patients and a restricted follow‐up period. Due to the limited number of patients, all patients in this study with indications for post‐and‐core restorations were treated with PEEK to better evaluate the survival rate of customized PEEK post‐and‐cores and related influencing factors. Therefore, no survival comparisons were made with other post‐and‐core restorations. Subsequently, randomized controlled trials, long‐term efficacy studies and possibly collaborate on multi‐center studies are required to provide more valuable evidence for post‐restoration.

## 5 Conclusion

Based on the results of this study, the following conclusions can be drawn:

1. The relative failure rate of the PEEK post‐and‐core restorations was similar to that of the other materials; however, the absolute failure rate was significantly lower. This suggests that PEEK post‐and‐cores have the potential to become a favorable material for obtaining reasonably satisfactory clinical results.
2. The amount of remaining tooth structure walls is significantly correlated with restoration success rates.

## Author Contributions

**Xin Wang:** methodology, investigation, writing–original draft. **Chen Liu:** investigation, data curation, formal analysis. **Yuchen Liu** and **Dan Ma:** conceptualization, methodology, validation, formal analysis. **Ruifang Ren, Mingxing Zhang,** and **Jiawen Guo:** methodology, writing–review and editing. **Yimin Zhao:** supervision, writing–review and editing. **Dongmei Li:** writing–review and editing, supervision. **Shizhu Bai:** conceptualization, writing–review and editing, supervision.

## Ethics Statements

To declare that this retrospective study conducted in Digital Center, School of Stomatology, The Fourth Military Medical University was conducted by the applicable ethical principles, including the 1964 World Medical Association Declaration of Helsinki and its later versions. It was also reviewed and approved (Approval Code: IRB‐REV‐2022188; Date: 01/12/2022) by the Ethics Committee of the Third Affiliated Hospital of the Fourth Military Medical University. In addition.

## Consent

Informed consent forms were obtained from all patients before the treatments.

## Conflicts of Interest

The authors declare no conflicts of interest.

## Data Availability

The datasets used and/or analyzed during the current study available from the corresponding author on reasonable request.
